# Brain-Derived Neurotrophic Factor-Mediated Neuroprotection in Glaucoma: A Review of Current State of the Art

**DOI:** 10.3389/fphar.2022.875662

**Published:** 2022-05-20

**Authors:** Lidawani Lambuk, Mohd Aizuddin Mohd Lazaldin, Suhana Ahmad, Igor Iezhitsa, Renu Agarwal, Vuk Uskoković, Rohimah Mohamud

**Affiliations:** ^1^ Department of Immunology, School of Medical Sciences, Universiti Sains Malaysia, Kota Bharu, Malaysia; ^2^ Department of Biosciences, Faculty of Science, Universiti Teknologi Malaysia, Johor, Malaysia; ^3^ Department of Pharmacology and Therapeutics, School of Medicine, International Medical University, Kuala Lumpur, Malaysia; ^4^ Department of Pharmacology and Bioinformatics, Volgograd State Medical University, Volgograd, Russia; ^5^ TardigradeNano LLC, Irvine, CA, United States; ^6^ Department of Mechanical Engineering, San Diego State University, San Diego, CA, United States

**Keywords:** brain-derived neurotrophic factor, glaucoma, neurodegeneration, neuroprotection, retina, retinal ganglion cell

## Abstract

Retinal ganglion cells (RGCs) are neurons of the visual system that are responsible for transmitting signals from the retina to the brain *via* the optic nerve. Glaucoma is an optic neuropathy characterized by apoptotic loss of RGCs and degeneration of optic nerve fibers. Risk factors such as elevated intraocular pressure and vascular dysregulation trigger the injury that culminates in RGC apoptosis. In the event of injury, the survival of RGCs is facilitated by neurotrophic factors (NTFs), the most widely studied of which is brain-derived neurotrophic factor (BDNF). Its production is regulated locally in the retina, but transport of BDNF retrogradely from the brain to retina is also crucial. Not only that the interruption of this retrograde transport has been detected in the early stages of glaucoma, but significantly low levels of BDNF have also been detected in the sera and ocular fluids of glaucoma patients, supporting the notion that neurotrophic deprivation is a likely mechanism of glaucomatous optic neuropathy. Moreover, exogenous NTF including BDNF administration was shown reduce neuronal loss in animal models of various neurodegenerative diseases, indicating the possibility that exogenous BDNF may be a treatment option in glaucoma. Current literature provides an extensive insight not only into the sources, transport, and target sites of BDNF but also the intracellular signaling pathways, other pathways that influence BDNF signaling and a wide range of its functions. In this review, the authors discuss the neuroprotective role of BDNF in promoting the survival of RGCs and its possible application as a therapeutic tool to meet the challenges in glaucoma management. We also highlight the possibility of using BDNF as a biomarker in neurodegenerative disease such as glaucoma. Further we discuss the challenges and future strategies to explore the utility of BDNF in the management of glaucoma.

## Introduction

Retinal ganglion cells (RGCs) are essential to processing perceived images, and their loss can lead to irreversible blindness, such as that seen in glaucoma (Gupta et al., 2016). Optic neuropathies, such as glaucoma, the second leading cause of blindness globally, are associated with the loss of RGCs and gradual degeneration of the optic nerve head (ONH); hence producing a characteristic pattern of visual field loss (Weinreb et al., 2018; Smith et al., 2020).

Glaucoma is a group of ocular disorders with multiple clinical phenotypes, but regardless of the subtypes, increased intraocular pressure (IOP) remains a widely recognized risk factor for the development and progression of glaucoma. Hence, currently lowering IOP to a target level is the only treatment option for glaucoma (Weinreb et al., 2014). Its etiology is unclear and what constitutes a “major contributor” to the disease development remains ambiguous. Numerous studies have been conducted to understand the pathophysiology of glaucoma and to identify the cellular and molecular targets for therapeutic intervention. A comprehensive review by Agarwal et al. (2009) stated that IOP elevation and vascular dysregulation remain the primary pathophysiologic factors, while the excitotoxic and the oxidative damage of the neurons are the secondary factor contributing to glaucomatous RGC loss (Agarwal et al., 2009).

Numerous investigators have documented the potential cytotoxic stimuli that contribute to the RGC death in glaucoma, including neurotrophin deprivation, glutamate excitotoxicity, mitochondrial dysfunction, glial activation, inflammation, endoplasmic reticulum (ER) stress, ischemia, and oxidative stress (Almasieh et al., 2012; Almasieh and Levin, 2017). From a therapeutic standpoint, each of these mechanisms could be a potentially attractive strategy for the intervention to achieve neuroprotection. Thus, neuroprotectants that can block the downstream cascades evoked by various cytotoxic stimuli have extensively been studied in an attempt to eradicate or slow down the optic neurodegeneration. The favourable effects of any of the currently investigated neuroprotective candidates, although observed in animal glaucoma models have not been replicated in clinical trials (Claes et al., 2019) and, in fact, the proposed benefits of some of these potential agents have now been challenged (Gribkoff and Kaczmarek, 2017). Besides, the consensus on the neuroprotective properties of potential therapeutic intervention, mode of its delivery also remains a challenge (Cvenkel and Kolko, 2020). Of note, poor drug delivery has always been one of the main concerns in the treatment of glaucoma, hence there is ever growing need for novel drug delivery systems. This includes the applications of nanotechnology-based formulations such as nano-fibre (Omer and Zelkó, 2021; Rohde et al., 2022), hydrogels (Lynch et al., 2020; Balla et al., 2022), contact lenses (Fan et al., 2020; Dang et al., 2022), and implants (Adrianto et al., 2021; Boia et al., 2022). Discussion on this area is beyond the scope of this paper. Comprehensive reviews by Akhter et al. and others (Akhter et al., 2022; Peng et al., 2022).

The RGC loss in glaucoma is accomplished through apoptosis irrespective of the initiating pathological stimuli (Munemasa and Kitaoka, 2013). Although the precise factors that contribute to glaucoma are still being debated, the neurotrophin deprivation theory, having arisen from the observed failing of the axonal transport, currently presents as one of dominant contributors. Neurotrophins are used in neuroprotective therapies because of their effective role in maintaining and improving the survival of neuronal cells (Jeanneteau et al., 2020). The deprivation of essential neurotrophins leads to induction of the apoptosis. Studies have shown that the neurotrophin-dependent mechanisms of cell death inhibition include the regulation of Bcl-2 and Bad proteins (Miller and Kaplan, 2001). The repetitive neuronal activity increases the secretion and action of neurotrophins at the synapses and modulates the synaptic transmission and connectivity (Schinder and Poo, 2000). Brain-derived neurotrophic factor (BDNF), a potent trophic factor, is predominantly expressed in the central nervous system (CNS) and is crucial for synaptic and structural plasticity. Its enhanced expression offers protection after injury (Feng et al., 2017).

BDNF exerts neuroprotective effects directly *via* the Tropomyosin receptor kinase B (TrkB) expressed in RGCs (Vecino et al., 2002; Osborne et al., 2018) and/or indirectly *via* the TrkB expressed in glia (Dekeyster et al., 2015a). In addition to the RGCs, the amacrine cells in the retina also produce BDNF, which can be transported retrogradely, from the brain to retina *via* axons (Cohen-Cory et al., 1996; Vecino et al., 2002; Grishanin et al., 2008; Harada et al., 2015). There is evidence that both the local synthesis and retrograde transport of BDNF, get reduced subsequent to excitotoxic insult causing changes in the synaptic dynamics, which in turn leads to retinal neurodegeneration (Quigley et al., 2000). In this review, the authors discuss the role of BDNF deficiency in the glaucomatous RGC loss. Many published studies describe the link between the lack of BDNF support to the RGCs as a trigger for their apoptosis (Ko et al., 2001; Vecino et al., 2002; Johnson et al., 2009; Shoeb Ahmad et al., 2013). Therefore, it is likely that aberrant BDNF expression and the underlying signaling pathways in the visual system paly a key role in the pathophysiology of glaucoma. Indeed, previous studies revealed that BDNF preserves the RGCs after the optic nerve axotomy in chronically hypertensive rats (Peinado-Ramón et al., 1996; Ko et al., 2001; Dahlmann-Noor et al., 2010). Similarly, there is a consensus on the association of central and local alterations in the BDNF-TrkB signaling pathway with the retinal or the optic nerve damage, indicating the role of BDNF in preserving the inner retinal elements. (Pease et al., 2000; Gupta et al., 2014; Dekeyster et al., 2015a). However, the different roles of the BDNF/TrkB signaling pathway in RGC versus other retinal neurons and glia cells have yet to be elucidated.

In glaucomatous eyes, BDNF expression was observed to be significantly lower in aqueous humor, lacrimal fluid, and serum relative to the healthy subjects (Almasieh et al., 2012), suggesting a possible correlation between low BDNF levels and the early stages of glaucoma (Ghaffariyeh et al., 2011). Considering involvement of multiple interacting mechanisms, blocking a specific pathway at the point of onset may not be adequate to stop pathological progression (Almasieh et al., 2012). Therefore, a focus on altering pathological cascade close to the merging endpoints may prove to be more meaningful to stop the RGC death.

In this review, the authors discuss the role of BDNF as a potential biomarker for the early detection of glaucoma. We propose BDNF-based neuro-repair as a novel strategy to complement neuroprotection achieved by the current treatments, focusing primarily on cell death and conferring continuous neurotrophic support after the initial injury. It is noteworthy that, despite significant advances, no neuroprotective agent that protects the RGCs from damage has shown benefits in clinical trials. Therefore, investigations into molecular and cellular events leading to the RGC death in glaucoma are warranted. The authors highlight the exogenous application of BDNF in the experimental model of glaucoma and its limitations when translating research findings into clinical application. Indeed, future studies conducted to better understand the critical role of BDNF and its signaling in healthy versus glaucomatous retinas will provide new insights that may prove to be essential as neuroprotective strategies to preserve RGCs. This review was carried out using the key words, brain-derived neurotrophic factors; retinal ganglion cells; neurodegeneration; neuroprotection; retina; glaucoma on PubMed, SCOPUS, and Web of Science databases. English language papers published from the year 1951, 1982 to 2022 are included in this review.

## Neurotrophic Factors

**FIGURE 1:**
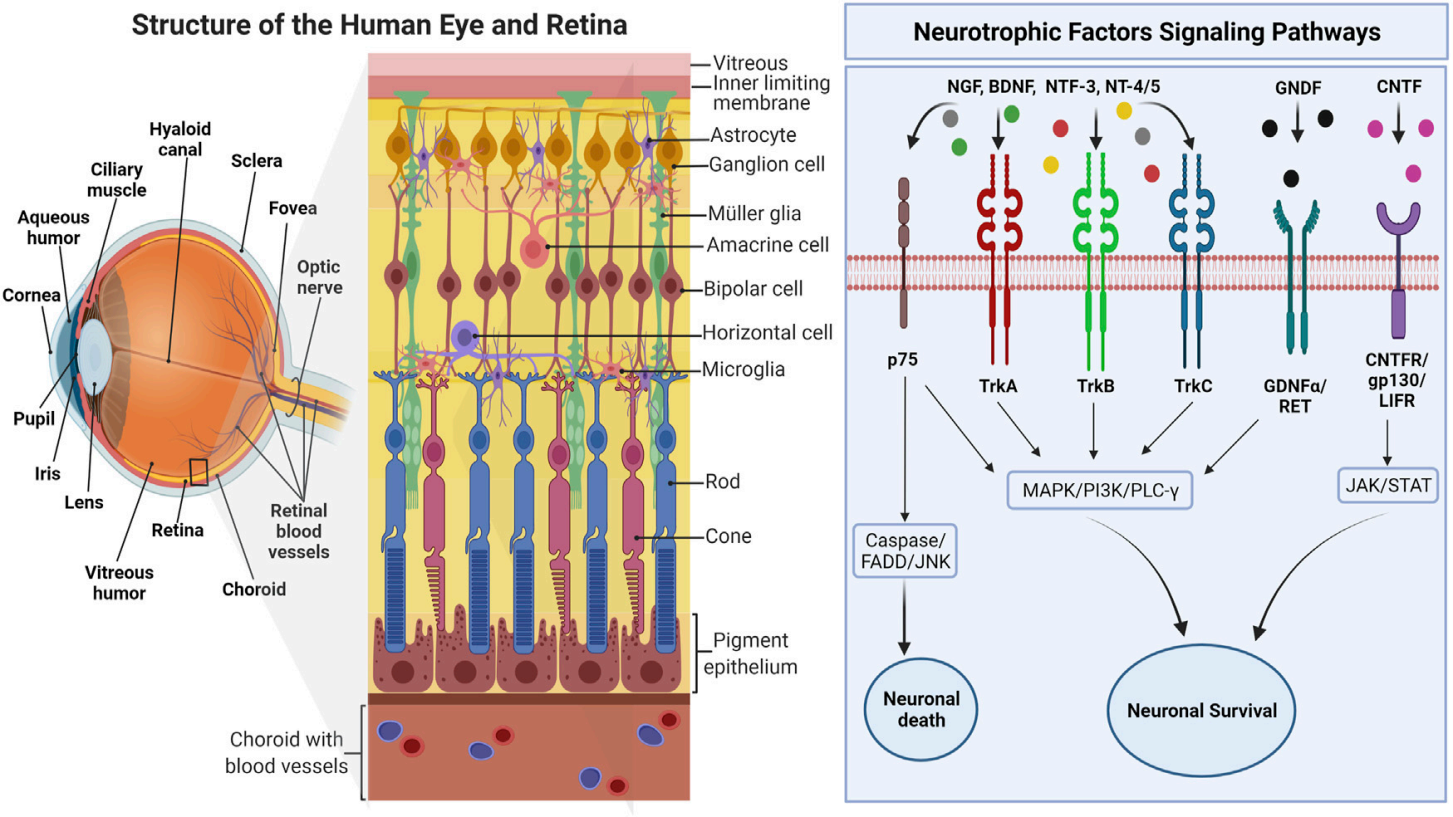
The overview of human eye anatomy with focus on the retinal structure. The right side of the figure depicts the interactions of various NTFs with major intracellular signaling pathways activated through corresponding receptors. Adapted from “Structure of The Retina” by BioRender.com (2022). Retrieved from https://app.biorender.com/biorender-templates.

Nerve growth factor (NGF) was the first growth factor identified in 1950s for its trophic (survival- and growth-promoting) effects on sensory and sympathetic neurons (Levi ‐ Montalcini and Hamburger, 1951). Later, in 1982, BDNF was discovered as the second member of the “neurotrophic” family of growth factors through isolation and purification from the pig brain. It was shown to promote survival of a subpopulation of neurons in dorsal root ganglion (Barde et al., 1982). Since the NGF and BDNF discovery, other members of the neurotrophin family have been described, such as neurotrophin-3 (NT-3), neurotrophin-4/5 (NT-4/5), ciliary neurotrophic factor (CNTF), and glial cell line-derived neurotrophic factor (GDNF) (Figure 1), each with a distinct profile of trophic effects on the subpopulations of neurons in the nervous system (Ip and Yancopoulos, 1996; Blum and Konnerth, 2005; Ibáñez and Andressoo, 2017). These molecules share several similarities, including their homologies in sequence, structure, and processing. They are synthesized as proneurotrophins, the immature precursors, and are converted to mature proteins after the proteolytic cleavage (Reichardt, 2006). These molecules bind to Tropomyosin receptor kinase (Trk) receptors and p75 neurotrophin receptor (p75NTR), and their affinity towards each of these receptors depends on their maturity (Lu et al., 2005). Mature Neurotrophic Factors (NTF) have a high affinity towards Trk receptors, which leads to cell survival and growth, while proneurotrophins have a high affinity towards p75NTR, which mainly elicits cell apoptosis. Each type of NTF binds selectively to specific Trk receptors: NGF binds specifically to TrkA; NT4 and BDNF activate TrkB; NT3 binds to TrkC, and all NF can bind to p75NTR (Reichardt, 2006). All the NTF-receptors bindings are not necessarily high affinity bindings. For example, the binding of BDNF to TrkB is of low affinity, but it can be changed when interacting with the Trk receptor and p75NTR (Chao, 2003). Upon activation, each receptor regulates several signaling pathways that are essential for neuronal development and function. Trk receptors regulate three major signaling pathways that mediate differentiation and survival, namely, mitogen-activated protein kinase (MAPK), phosphatidylinositol 3-kinase (PI3K), and phospholipase Cγ (PLC-γ).

p75NTR with the intracellular death domain, similar to that in tumor necrosis factor receptors (TNFR), regulates survival and inflammation through nuclear factor kappa B (NF-κB), neuronal apoptosis through Jun-N terminal kinase (JNK), and reduced growth cone motility through RoA/ROCK signaling pathway (Huang and Reichardt, 2003). However, these two types of receptors could interact with each other or another type of receptor and transduce different binding affinities and signaling pathways that further contribute to additional functions of NTF and its receptors (Chao, 2003). Apart from forming complexes that produce high-affinity binding sites for NTF, activation of the Trk signaling pathway, such as NF-ΚB, by p75NTR has shown a synergistic contribution to neuronal survival (Huang and Reichardt, 2003). Although proapoptotic signaling of p75NTR is suppressed by Trk signaling, primarily through PI3K, this interaction is not always fully efficient, given that the crosstalk of p75NTR, and Trk signaling could also induce apoptosis in the presence of ceramide and regulate the number of mature cells (González-Hoyuela et al., 2001). The specificity of Trk receptors to each NTF is regulated by the presence or absence of an insert, a brief sequence of amino acids in the juxtamembrane region (Huang and Reichardt, 2003). For instance, TrkB without the insert can be activated by BDNF only, whereas the presence of the insert on TrkB makes it activable by NT3 and NT4 as well (Yacoubian and Lo, 2000). Hence, the receptors, either full length (TrkB-FL) or truncated (TrkB-Tc), would regulate distinct features of the NTF signaling. Compared to other NTFs, BDNF is highly expressed in the adult brain, mainly in the hippocampus, and is tightly regulated by neural activity. Apart from neuronal survival, BNDF is widely accepted to play a critical role in synaptic plasticity and memory (Sasi et al., 2017).

## BDNF and Its Receptors

The broad range of functions served by BDNF owe to the complexities of neurotrophin production, secretion, and receptor signaling in the nervous system. Once secreted, BDNF can be activated in two forms: prodomain of BDNF (cleaved precursor protein; pro-BDNF) and mature BDNF (mBDNF), which exert their functions primarily through p75NTR and sortilin signaling and TrkB and p75NTR/TrkB, respectively (Kutsarova et al., 2021). Although mBDNF has a high affinity of binding to TrkB, it binds to p75NTR when the expression level is aberrant, hence stimulating signaling cascades in the manner opposite to the TrkB receptor. Because of the opposing affinities, the intra-/extracellular cleavage of BDNF becomes another critical factor in regulating the downstream signaling effects of BDNF (Lee et al., 2001).

The Trk receptors dimerize in response to a ligand binding and autophosphorylate. There are several isoforms of TrkB, and the most abundantly expressed are the full-length (TrkB-FL) and the truncated (TrkB-Tc) forms. TrkB-Tc lacks an intracellular kinase domain (Eide et al., 1996), hence it functions as a dominant-negative receptor, forming heterodimers with full-length receptors and blocking neurotrophin signaling. In astrocytes and Schwann cells, the truncated form has been suggested to regulate the pool of neurotrophins and keep them from degrading or signaling until they are released into the extracellular space (Alderson et al., 2000). The pro-death receptor, p75NTR, comprises a cytosolic death domain that is highly expressed during development (Dechant and Barde, 2002). It acts canonically by mediating both pro-death and pro-survival signals, which depend entirely on associations with cytoplasmic proteins (Dechant and Barde, 2002). In contrast, several intracellular tyrosine residues of TrkB-FL can be phosphorylated (Huang and Reichardt, 2003). Because of this, the three signaling cascades (i.e., MAPK, PI3K, and PLC-γ pathways) promote and govern the activity-dependent and tissue-specific expression of BDNF (Chen et al., 2003). The promoters translocate to the nucleus, where transcription of mRNAs responsible for producing the heterogeneous population of BDNF occurs (Minichiello, 2009). The BDNF mRNA splice variant has been described in multiple species (Dekeyster et al., 2015a). Of importance, environmental experiences such as stress-induced epigenetic modifications can influence the BDNF gene activity and epigenetic marking of the BDNF gene (Roth and Sweatt, 2011).

Since these receptor-mediated actions are thought to act contradictory, the dynamics may help in balancing the growth and death of neurons. Of note, preferentially, pro-BDNF signaling through presynaptic p75NTR is essential for axonal retraction in growing neuromuscular synapses and results in antigrowth signaling (Lee et al., 2001). The modulator, pro-BDNF, would selectively promote N-methyl-D-aspartate (NMDA) activity, along with glutamate, through p75NTR. Unlike pro-BDNF, mBNDF *via* presynaptic TrkB leads to axonal stabilization and results in pro-growth signaling (Je et al., 2013). Mature BDNF-TrkB signaling mediates long-term potentiation (LTP) through pre- and post-synaptic mechanisms, such as by influencing local protein synthesis, spine remodeling, or gene transcription (Park and Poo, 2012). Since BDNF is predominantly secreted as pro-BDNF, proteins that cleave the prodomain may influence which receptors are triggered by the BDNF release, giving yet another regulatory mechanism for BDNF signaling (Chen et al., 2005). BDNF-TrkB signaling can act as both mediator and modulator for plasticity-inducing neuronal activity. Moreover, BDNF with neurotransmitter signaling released within a critical time window can act as the instructor for immediate synaptic plasticity. This is why BDNF has been of interest as a stimulant for protective and restorative treatments in both neurological and psychiatric disorders. What is also known about BDNF is that compared to other NTFs, the former is the superior factor for the RGC survival under glaucomatous conditions (Almasieh and Levin, 2017). This has been proven by the exogenous application of BDNF in the developing retinotectal system where the RGC axons showed arborization and growth, contrariwise to the depleted endogenous BDNF, which hampered presynaptic trafficking and axonal branch stabilization (Kutsarova et al., 2021).

## BDNF in Retina

Emerging evidence on the importance of BDNF as a neurotrophin in addition to NGF, was discovered by Barde et al. (1982). In this seminal study, BDNF was shown to exert trophic effects in the survival of a subpopulation of dorsal root ganglion neurons and the fiber growth in cultured embryonic chicks (Barde et al., 1982). The same effect was later identified in the adult human brain, where a sustained expression of BDNF was associated with increased number of receptors specific to dendrite growth indicating stimulation of neurogenesis and perhaps appearance of new neurons (Tyler et al., 2002). Importantly, BDNF is required for development and survival of dopaminergic, GABAergic, serotonergic, and cholinergic neurons. Indeed, an in-depth interpretation of the effects of BDNF on the development and survival of retinal neurons may provide more significant insights into role of BDNF/TrkB pathway in the pathogenesis and, ultimately, loss of RGCs.

Unlike other retinal neurons, the axons from RGCs project to various areas of brain *via* the optic nerve (Crair and Mason, 2016). Much of the information regarding projection of optic nerve fibres in brain has been gathered from studies involving animals like rodents, chicks, and tadpoles (*Xenopus*). However, the significant difference between these experimental species and humans is the distribution of RGC axon projections in brain. In higher mammals, such as macaque, the most RGC projections synapse in the lateral geniculate nucleus (LGN), with fewer axons extending to the superior colliculus (SC) (Perry et al., 1984). However, in other experimental species such as mice, 85%–95% of the RGCs project to the SC (Ito and Feldheim, 2018; Reinhard et al., 2019). The development of retinal axons and their projections undergoes changes over a broad time frame to regulate the structural morphology and connectivity. Most prominently 50% of RGCs undergo apoptosis during pre- and early postnatal period (Guerin et al., 2002). BDNF exhibits a spatiotemporal expression at this stage, which may play an essential role in maintaining the growth of the neuroretina as well as other structures of eye such as cornea, lens, trabecular meshwork, and ciliary body (Bennett et al., 1999).

As Frost et al. (2001) reported, while the BDNF protein expression in the hamster SC declines significantly to attain adult level by postnatal day 15, the same in retina increases significantly to attain adult level at the same time point. In SC, the BDNF protein level increases only during RGC branching but plateaus by the time arborization nears completion and then declines once the adult level is reached on the postnatal day 18 (Frost et al., 2001). It is clear that the relationship between the enhanced BDNF level and the neuronal activity of developing RGCs impacts their survival into adulthood. Likewise, when the RGC axons arborize in the SC, mature BDNF levels rise (Frost et al., 2001). Multiple events in the anatomical and functional maturation of the hamster retinal projection system are temporarily linked with developmental changes in retinal and SC BDNF protein concentrations. Moreover, BDNF expression is activity-dependent during the period of RGC death and synapse formation (Cohen-Cory and Lom, 2004). Both BDNF and TrkB mRNA and protein are expressed in the retina and SC at this time and are exceptionally high in RGC target sites (Perez and Caminos, 1995; Cohen-Cory et al., 1996; Frost et al., 2001; Cohen-Cory and Lom, 2004).

BDNF can be locally produced by RGCs and astrocytes in the retina and is transported to target areas *via* paracrine and autocrine actions (Cohen-Cory et al., 1996). However, it is still debatable whether BDNF/TrkB support of RGC survival throughout the development is due to retrograde or anterograde transport or the retinal BDNF sources persist into adulthood. What is undisputable is that BDNF promotes neuronal survival, axonal guidance and regulates the excitatory and inhibitory synaptic transmission in the visual system (Tyler et al., 2002). Indeed, BDNF was the major player of activity-dependent branching within the SC and remodeling of the RGC axons arborization (Marler et al., 2014). Owing to its highly regulated expression due to transcription, translation, and post-translational modifications (Ruiz et al., 2014), BDNF is believed to modulate critical protein synthesis in activity-dependent synaptic plasticity (Pollock et al., 2001). This complex regulation demonstrates the vitality and diversity of BDNF in supporting existing neurons after cellular insults in multiple neurodegenerative diseases (Pollock et al., 2001). For instance, reduced expression of BDNF and polymorphism -are closely associated with Alzheimer’s disease (AD) progression (Kunugi et al., 2001; Beeri and Sonnen, 2016). A meta-analysis reported that serum BDNF decrease in individuals affected by Parkinson Disease (PD), supporting the association of reduced BDNF level and PD (Jiang et al., 2019). The same was documented in the person with relapse-remitting multiple sclerosis, where BDNF concentration was significantly lower than in healthy individuals (Wens et al., 2016).

## NTF in Glaucoma

It is now well established from various experimental glaucoma studies that NTFs effectively promote the survival of neurons and prevent apoptotic ganglion cell death (Mallone et al., 2020). This was supported by the finding that the therapies that significantly preserved the RGCs in the rat model of glaucoma were associated with elevated BDNF expression as compared to the untreated controls (Martin et al., 2003). Further cementing the significance of BDNF as a neuroprotective agent, it has been observed that exogenously applied BDNF inhibits the RGC loss and optic nerve damage in various acute and chronic glaucoma models (Gao et al., 2002; Mohd Lazaldin et al., 2020; Fudalej et al., 2021). Similar evidence of reduced RGC loss was also observed in response to the topical application of NGF (Colafrancesco et al., 2011; Lambiase et al., 2011). Topically administered NGF rescued RGCs from degeneration and enhanced the visual function of individuals with advanced glaucoma (Lambiase et al., 2011). Interestingly, in a case-control study, serum BDNF and NGF levels were low in patients with early and moderate glaucoma, indicating that the NTFs have a potential to serve as diagnostic biomarkers for glaucoma (Oddone et al., 2017). The overexpressed CNTF has also been shown to exert a strong protective effect on RGCs in an experimental rat model (Pease et al., 2009). In an ocular hypertension-induced rat model of glaucoma, the administration of CNTF resulted in substantial reduction of the RGC loss, suggesting that CNTF promotes the survival of RGCs (Ji et al., 2004). CNTF has also been shown to promote regeneration in various retinal degeneration models (Li et al., 2010; Wen et al., 2012). Despite the evidence supporting the neuroprotective effects, the use of NTFs is challenging in clinical settings due to difficulties in their passage *via* anatomical barriers, such as, the blood-brain barrier, the blood-retinal barrier and the blood-aqueous barrier. Moreover, the challenges posed by their short half-lives and wide-ranging effects requires target-specific formulations (Mallone et al., 2020).

## BDNF Deprivation and Its Link to Glaucoma

One of the hypotheses proposes that the hindered defense mechanism of RGCs stems from the compromised neurotrophin transport to the cell bodies (Munemasa and Kitaoka, 2013; Guo et al., 2020). Since neurotrophins, particularly BDNF, are transported to the retina primarily in a retrograde manner, the transport blockade prevents BNDF synthesized locally, in soma and dendrites of neurons, to bind to the Trk receptors at the axon terminals (Dekeyster et al., 2015a). The lack of trophic support to RGCs may trigger apoptotic signalling and resulting in RGC loss (Kimura et al., 2016). In theory, BDNF deprivation in RGCs exerts stress, which triggers the cellular apoptotic pathways via JNK-mediated signaling, resulting in activation of proapoptotic BCL-2 family of proteins and leading to mitochondrial dysfunction. As a response to disease or injury, RGCs are known to upregulate the BDNF gene expression to circumvent apoptosis signaling and support the survival of the remaining RGC population. The same trend can be seen in axonal growth rate; however, it occurs only within the axonal terminals (de Rezende Corrêa et al., 2015). Apart from the RGCs, the inner retinal cells and photoreceptors are responsive to BDNF, implying that the neurotrophins are locally synthesized in the inner nuclear layers (Perez and Caminos, 1995). TrkB is highly expressed in RGCs, amacrine, and Müller cells, suggesting that the cellular target of the trophic action of BDNF is in the inner retinal elements (Zhang et al., 2005; Weber et al., 2010).

**FIGURE 2:**
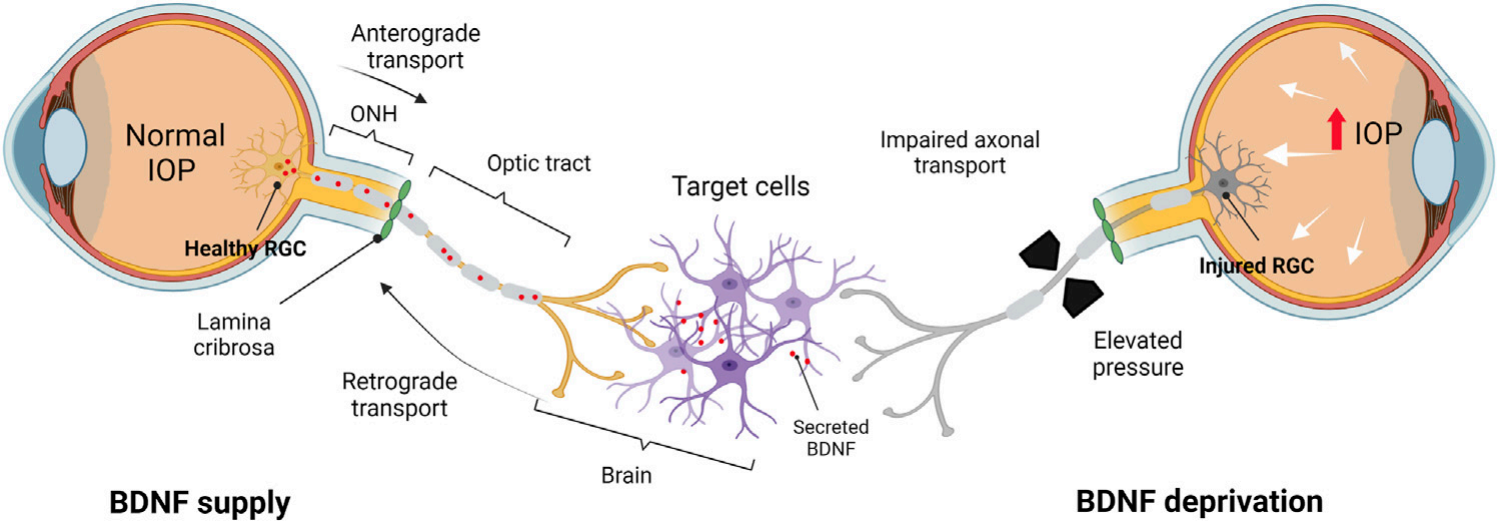
BDNF deprivation theory in the adult human visual system. BDNF supply: in healthy conditions, BDNF is synthesized and secreted by cells in the brain and transported retrogradely *via* the optic nerve toward the somas (RGCs). BDNF deprivation: axonal transport is perturbed due to the elevated IOP and RGCs are deprived of target-derived (brain) BDNF, leading to the stressed RGCs undergoing apoptosis. IOP, intraocular pressure; ONH, optic nerve head. Created by Biorender.com (https://biorender.com/).

Considering the role of neurotrophins in RGC survival, dampening the endogenous stimuli, especially during episodes of insult, leads to substantial RGC damage (Figure 2). Deficient BDNF-TrkB signaling has been shown to be associated with RGC loss in various studies (Pease et al., 2000; Quigley et al., 2000; Iwabe et al., 2007; Osborne et al., 2018). Quigley et al. (2000) demonstrated that acute IOP elevation substantially suppresses the retrograde BDNF delivery to the ONH from the SC in adult rats, contributing to neuronal loss due to BDNF deficits. This has been attributed to the aberrant distribution of the axoplasmic transport of the trophic factors from target neurons in the SC and dLGN (Pease et al., 2000; Tanaka et al., 2009). Similarly, Pease et al. (2000) reported that the obstructed retrograde transport of BDNF gives rise to abnormal TrkB axonal distribution, focal accumulation of TrkB and BDNF, increased levels of TrkB in GCL, and increased TrkB in glia (Pease et al., 2000).

Multiple *in vivo* studies have suggested that the deficits of BDNF expression mark the RGC damage in glaucoma, and its interrupted axonal transport has been implicated in the progressive development of optic neuropathy in experimental models of glaucoma (Gupta et al., 2014; Feng et al., 2016; Osborne et al., 2018; Chitranshi et al., 2019; Wójcik-Gryciuk et al., 2020; Conti et al., 2021; Lazzara et al., 2021). The BDNF axonal transport in injured RGCs analysed *via* live-cell imaging was shown to exhibit reduced activity before the death of RGCs (Takihara et al., 2011). This finding is consistent with that in glaucomatous human eyes (Ghaffariyeh et al., 2009; Ghaffariyeh et al., 2011; Gupta et al., 2014; Shpak et al., 2018; Igarashi et al., 2021) as BDNF deficits were detected in serum (Ghaffariyeh et al., 2011; Shpak et al., 2018), aqueous humour (Shpak et al., 2018; Igarashi et al., 2021), and lacrimal fluid (tears) (Ghaffariyeh et al., 2009; Shpak et al., 2018) of patients with early glaucomatous changes. Although it stimulates the expression of BDNF and its receptors, excitotoxicity induced by NMDA may also alter the retrograde transport of BDNF in the optic nerve and deprive it of the neurotrophins (Lambuk et al., 2017). It is also noteworthy that upon acute insult, the expression of BDNF-TrkB in the mouse retina is enhanced above the normal levels with extended axon survival (Feng et al., 2017). It is also hypothesized that the neuronal compartments, including the soma, axon terminal, and dendrites, appear to start the orchestration of BDNF-TrkB signaling differently (Chowdary et al., 2012). Manipulating the RGC target regions in which the signal is initiated may be a way of preventing the RGC death and delaying the progression of glaucoma or.

While the interruption in the retrograde transport is present at the early phase of the damage, the BDNF protein is synthesized rapidly in RGCs as an endogenous neuroprotective response, corroborating the idea of locally produced vs. retrogradely transported BDNF (Vecino et al., 2002). In addition, BDNF and TrkB is abundantly expressed in RGCs after axotomy, indicating that the endogenous protective response may contribute to the short-term survival of the neurons (Hirsch et al., 2000). Similar to BDNF, TrkB may be transported and stored at the axon. The intense and consistent TrkB expression was detected in the nerve fiber layer (NFL) post optic nerve lesion in the adult rat retina (Cui et al., 2002). In short, TrkB receptors could be synthesized in the soma and transported anterogradely or isolated at the nerve terminals and retrogradely transported to the soma of RGCs. However, they are not likely to promote long-term survival of the cells due to the reduced expression of BDNF expression subsequent to initial upregulation in RGCs. The limited presence of BDNF could also be attributed to the metabolic changes in injured neurons (Hu et al., 2010).

It is also argued that interrupted retrograde BDNF delivery can not be considered as the only cause of RGC death in glaucoma. This argument is supported by the observation that the adult porcine RGCs *in vitro* continued to survive and maintain their regular interaction with neighbouring neurons despite the lack of exogenous BDNF and dissociation from target tissues and presynaptic inputs (García et al., 2003). BDNF is also anterogradely transported to the CNS, where it serves as a survival factor for postsynaptic neurons in the SC and dLGN (Caleo et al., 2000).

### Axonal Transport

It is suggested that RGC axonal transport alterations are a critical pathological component concomitant with the early increase in the IOP. Anterograde axonal transport delivers proteins, lipids, and mitochondria to the distal synapse (Fahy et al., 2016). Since neuronal proteins and molecules are predominantly synthesized in the cell body, the long axon hinders soma-derived proteins from reaching their presynaptic destinations at the axonal terminals (Chowdary et al., 2012). However, anterograde transport is the vital means of transferring newly generated synaptic proteins, ion channels, lipids, and mitochondria to their axonal destinations (Chowdary et al., 2012). Conversely, retrograde axonal transport from the axon to the soma is involved in the transport of waste substances, for instance, degraded molecules and organelles for clearance (Guo et al., 2020). This axonal transport also serves as a channel for the intracellular transport of distal chemical and biological trophic signals back to the cell body. Alongside downregulated RGC-specific genes and metabolic changes, functional and mechanical impairment of the retrograde axonal transport can be an early indicator of glaucomatous damage (Vidal-Sanz et al., 2012). The failure could result from the distortion of the elements, including defects in the cytoskeletal filaments and motor protein, which is the key to the axonal traffic machinery (Perlson and Holzbaur, 2007). It may impact the delivery of factors essential for the cell survival and the retinal function (Lu et al., 2014; Fahy et al., 2016). Indeed, this idea corroborates the neurotrophin deprivation theory as one of the mechanisms of RGC loss (Fahy et al., 2016).

The anterograde axonal transport from RGCs may occur for both the endogenous and exogenously administered BDNF (Caleo et al., 2000; Caleo et al., 2003; Butowt and von Bartheld, 2005). A fraction of BDNF transported *via* the anterograde path is newly produced by RGCs or the neighbouring retinal cells (Butowt and von Bartheld, 2001). Several attempts have been made to show that the role of BDNF secreted and delivered from RGCs in an anterograde direction along axons is to promote survival factors for post-synaptic neurons after retinal injury (Caleo et al., 2003; Dengler-Crish et al., 2014). During the development of rodents, deficits of retinal BDNF-TrkB signaling retracted the RGC axons from the dLGN and affected the inner retinal neuronal circuit development, implying that the retrograde transport blockade in the retina does not affect the retinogeniculate connectivity (Menna et al., 2003; Grishanin et al., 2008). RGCs also continuously deliver BDNF to the SC in adulthood (Avwenagha et al., 2006). In adult rats RGCs express BDNF-TrkB post-axotomy, supporting the idea that the survival of damaged RGCs may depend on the sufficient BDNF levels (Vecino et al., 2002).

The overproduction of BDNF in RGCs ensures fine-tuning of proper target innervation in the visual cortex of the brain, including the dLGN, SC, the suprachiasmatic nucleus, and the pretectum (Leinonen and Tanila, 2018). Inadequate trajectory to these target areas could be due to the insufficient endogenous retinal BDNF or perturbed axonal transport of the neurotrophin (Johnson et al., 2009). BDNF is also synthesized in the SC, the primary target of optic projections, and can be delivered to the retina retrogradely *via* RGC axons (Spalding et al., 2004). These pieces of information highlight that BDNF is excessively produced after the onset of target innervation and at the early stages of RGC development. However, adult RGCs are supported by BDNF, which is primarily produced locally (Beros et al., 2021). The enhanced retrograde transport of BDNF is triggered when the local trophic support is progressively interrupted. In theory, although local intra-retinal BDNF supplies may be delivered anterogradely for long-term survival, RGCs are eventually considered to be dependent on the competition for limited amounts of retrograde BDNF support (Beros et al., 2021). Further studies are needed to explore this support of RGC survival by BDNF during the development and adulthood when experiencing injuries or stress.

The obstruction of BDNF delivery and accumulation of TrkB at the ONH plays a vital role in the pathogenesis of glaucoma (Pease et al., 2000). In animal models with elevated IOP, the retrograde transport of BDNF-TrkB was blocked at the ONH contributing to BDNF deficits eventually leading to gliosis and neuronal loss in the retina (Quigley et al., 2000; Gupta et al., 2014). Similar observations were made by Dekeyster et al. (2015a) in the mouse model of optic nerve crush due to blockage of the retrograde delivery of BDNF. Temporary upregulation of retinal BDNF-TrkB after injury suggests that it acts as a natural protection mechanism to overcome neurotrophin transport deficits (Gao et al., 1997; Johnson et al., 2009; Dekeyster et al., 2015a).

Interestingly, in mice, the absence of BDNF did not affect the number of RGCs in the mature retina (Chitranshi et al., 2019; Beros et al., 2021). Noteworthy, that for the target-dependent survival during the early retinal development, BDNF-TrkB signaling is not required, whereas in adult animals, it intensifies to reduce RGC degeneration in the presence of pathogenic stimuli. However, exogenous administration of BDNF to the optic tectum of developing *Xenopus* and chick improved the RGC dendritic arbor complexity (Lom and Cohen-Cory, 1999; Cohen-Cory and Lom, 2004). In fact, during the RGC development BDNF can be said to affect the RGC and optic tectum architecture differently depending on its source and transport. Despite the elegant series of studies, the interplay between retrograde and anterograde BDNF axonal transport in the human retina remains unclear. Most studies observed that BDNF differentially modulates the survival of RGCs in rodents, and there remains a need to investigate the same effects in humans.

## BDNF as a Neuroprotective Agent

Neuroprotection is an ideal therapeutic approach in glaucoma to keep RGCs alive (Almasieh and Levin, 2017). The goal of neuroprotection in glaucoma is to preserve the optic nerve independent of the IOP reduction and thus prevent or delay the RGC apoptosis and axonal degeneration (Tsai, 2020; Shalaby et al., 2021). Hence, any protective intervention that directly aims at promoting the health and survival of RGCs has the potential as an antiglaucoma agent. From this standpoint, BDNF seems an attractive option for further investigations.

As highlighted earlier, BDNF increases the number of receptor sites in neurons that lead to dendrite and axonal growth and stimulate neurogenesis (Miranda et al., 2019). It is required for both the development and survival of dopaminergic, GABAergic, serotonergic, and cholinergic neurons (Park and Poo, 2012). The cellular basis for learning and memory rests with the synapses within the hippocampus. The activation of the BDNF associated TrkB intracellular pathway was shown to improve cognition, which correlated with an increase in the synaptic density (Castello et al., 2014). Accordingly, the upregulation of both BDNF and TrkB was detected in the brain areas with neuronal plasticity. Because of this relationship, BDNF is considered a molecular mediator for regulating the synaptic plasticity, playing a pivotal role in memory formation and consolidation (Zeng et al., 2012). Disruption of the pathways that transport and produce BDNF can cause clinical symptoms of deteriorating memory and cognitive dysfunction (Leal et al., 2017). Clinical studies have shown a causal relationship between lower levels of BDNF and cognitive decline observed with aging, schizophrenia, and Rett syndrome (Zuccato et al., 2011; Autry and Monteggia, 2012; Soares et al., 2016).

In animal models of glaucoma, disrupted BDNF axonal transport was observed, and BDNF injection into the superior colliculus (SC) of neonatal hamsters resulted in a 13–15-fold reduction in RGC pyknosis, showing that the BDNF plays a significant role in promoting the RGC survival (Ma et al., 1998). In addition, clinical and experimental studies have shown that the BDNF/TrkB complex is downregulated in the inner retina and the optic nerve head of glaucomatous eyes (Pease et al., 2000; Gupta et al., 2014). TrkB also gets gradually downregulated in response to the neuronal damage, suggesting it may be less responsive to the BDNF levels (Shen et al., 1999). Compared to the control group, BDNF levels in the blood of primary open-angle glaucoma patients and in the tears of normal-tension glaucoma patients were significantly lower (Ghaffariyeh et al., 2011; Oddone et al., 2017). A study by Rudzinski et al. (2004) showed that changes in the retinal expression of neurotrophic factors significantly correlate with the RGC death. The same study observed a transient upregulation of both retinal NGF and TrkA receptors after 7 days of ocular hypertension (Rudzinski et al., 2004). The sustained upregulation of retinal BDNF after 28 days of ocular hypertension was also recorded. However, the expression of TrkB receptors as well as NT-3 levels remained unchanged; although, there was an early and sustained increase of TrkC receptors in Müller cells, but not in RGCs (Rudzinski et al., 2004). Thus, the asymmetric upregulation of neurotrophin and its receptor patterns may suggest that the dysregulated activity of neurotrophic factors plays a role in the RGC apoptosis.

Other studies also provide convincing evidence about the neuroprotective effects of BDNF in retina. Ma et al. (1998) showed a reduction in the RGC death after BDNF was injected into the SC (Ma et al., 1998). Studies by Pease et al. (2000) using an experimental model of glaucoma in monkeys and rats with acute IOP elevation showed that the BDNF transport in the optic nerve head was dysregulated (Pease et al., 2000). However, once BDNF was injected *via* an intravitreal injection, it reduced the RGC degeneration (Mansour-Robaey et al., 1994). Ko et al. (2001) also found that the injection of BDNF to rats with elevated IOP increased the survival rate of RGCs as compared to the untreated animals (Ko et al., 2001). Exogenous, topical, or intravitreal BDNF was found to be potent in activating the pro-survival signaling pathways in RGCs following induction of ocular hypertension in experimental animals (Ma et al., 1998; Ji et al., 2004). In another study, recombinant human BDNF eye drops caused recovery of the pattern electroretinogram (P-ERG) and the visual cortex evoked potential (VEP) damage (Domenici et al., 2014) in the presence of chronic intraocular hypertension. As measured by the Brn3 immunopositive cell density in the RGC layer using retinal immunocytochemistry, this was linked to an increase in the RGC survival (Domenici et al., 2014). In addition, three consecutive intraocular injections of BDNF at 1.0 g/L in moderately chronic hypertensive rat eyes resulted in a 2-week increase in the RGC survival with no cumulative effect (Ko et al., 2001). High-dose BDNF, when injected intravitreally in animal models of the optic nerve injury, induced a rapid and considerable downregulation of TrkB expression, reducing the BDNF efficiency (Chen and Weber, 2004). On the other hand, it was shown that cyclic AMP (cAMP) induced neuronal sensitivity and axonal regeneration in BDNF-treated culture. Enhanced survival was associated with the increased availability of TrkB (Park et al., 2004). This allowed more TrkB to bind to BDNF on the surface of RGCs, enhancing the cell survival. By combining the treatment with forskolin, a TrkB agonist, the cAMP level gets elevated and the responsiveness of RGCs to BDNF is enhanced (Hu et al., 2010). This may imply that injured RGCs are less active and more sensitive to BDNF, which may affect their overall activity more than that of their healthy counterparts. Furthermore, studies have shown that increased BDNF/TrkB expression might harm neuronal homeostasis by increasing the glutamate excitotoxicity (Maki et al., 2015). TrkB activation has been shown to accelerate the glutamate-induced mortality in rat neuroblastoma cells (Maki et al., 2015), and higher BDNF levels have been reported in muscles of amyotrophic lateral sclerosis (ALS) patients.

Use of high-dose subcutaneous or intrathecal rhBDNF in patients with ALS did not provide neuroprotective effect (Ochs et al., 2000). As a result, several efforts have been made to target TrkB specifically with low molecular weight substances. A flavonoid-based TrkB agonist, 7,8-dihydroxyflavone (7,8 DHF) has been tested for this purpose. In an animal model of Parkinson’s Disease and in an *in vitro* model of excitotoxic and oxidative stress-induced RGC apoptosis, this compound proved effective in activating TrkB downstream signaling and exerting neuroprotective effects (Jang et al., 2010; Gupta et al., 2013). Distinct TrkB agonist antibodies have enhanced the RGC survival *in vitro* and *in vivo* in acute and chronic glaucoma models (Bai et al., 2010; Hu et al., 2010). A cell-permeable phosphine–borane compound promoted the RGC protection in a rat model of optic nerve injury by stimulating the ERK1/2 pathway to directly activate the survival signaling downstream of TrkB (Almasieh et al., 2011). However, further research is needed to weigh the benefits and the drawbacks of activating BDNF/TrkB signaling pathway in the management of neurodegenerative diseases.

## Current Status

BDNF plays a role in a myriad of pathophysiologic pathways (TGF-*β*, MAP kinase, Rho kinase, JNK, PI-3/Akt, PTEN, Bcl-2, Caspase, Calcium-Calpain) and could serve as a promising candidate for devising therapies to enhance RGC survival in glaucoma (Chitranshi et al., 2018). Various studies targeting the BDNF-TrkB signaling pathways, primarily through topical or intravitreal applications, have shown BDNF to impede the RGC loss effectively in animal models. Indeed, NTFs have been a subject of interest for neuroprotection in the past two decades due to their pivotal roles in maintaining and enhancing neuronal survival (Poo, 2001; Mandolesi et al., 2005). Although, in the glaucomatous retina, BDNF and its receptor showed no distinct differences in the expression levels as compared to the normal retina, the treatment with topical drugs, such as prostaglandin analogs, caused an increase in the expression of BDNF and TrkB (Harper et al., 2020). The studies, however, have also demonstrated that the effect of the BDNF treatment on the RGC survival is short-lived, whereas the repeated or over-exposure decreased the responsiveness or even desensitized TrkB activation by BDNF (Klöcker et al., 1998; Dekeyster et al., 2015b).

Although, some agents have shown promising results in preclinical studies, most are not ready for application in human trials. Some of these agents such as alpha 2-agonist brimonidine (BMD) (Lafuente et al., 2002), NMDA receptor antagonist (memantine) (Weinreb et al., 2018), ciliary neurotrophic factor (CNTF) (Kauper et al., 2012), rhNGF (Gala et al., 2021) or nicotinamide (vitamin B3) (Hui et al., 2020) have advanced to comprehensive randomized controlled trials (Osborne et al., 2018; Lee et al., 2012). However, the results of some of these trials are not favourable (Kolko, 2015). For example, BMD, commonly prescribed in clinics to reduce the IOP, was found to improve the BDNF production and preserve RGCs when administered systematically to mouse and rat models of IOP-independent glaucoma (Lee et al., 2012; Lee et al., 2010; Metoki et al., 2005). The neuroprotective effect of BMD was also shown in ocular hypertensive animals and those with optic nerve injury in terms of prevention of visual defects (Kitaoka et al., 2015; Semba et al., 2014). Clinically, monotherapy with 0.2% BMD tartrate causes significantly greater reduction in the progression of visual field defects compared to 0.5% timolol maleate eye drops in a 30-months Low-Pressure Glaucoma Study group trial (ClinicalTrials.gov identifier NCT00317577) (Krupin et al., 2011). However, the report raised concerns because the progression rate in the individuals treated with timolol was worse than that in the untreated group as observed in other trials, such as the Collaborative Normal Tension Glaucoma Study, suggesting that timolol enhances the disease progression rather than BMD reducing it, or it could be a combination of the two (Group, 1998). Besides, topical administration of BMD is associated with greater side effects, including hyperemia, discomfort, and hypersensitivity, as compared to other topical anti-glaucoma medications. A selectively higher dropout rate could have skewed the results in the BMD arm as compared to the timolol arm (Storgaard et al., 2021). Unfortunately, this observation is not limited to BMD only. None of the completed double-blind clinical trials for NTF administrations have met predefined endpoints for clinical efficacy (Shen et al., 2021). The overall progress of clinical trials demonstrated findings that could raise the uncertainty caused by ocular drug delivery challenges, thus highlighting the need to develop more relevant and appropriate clinical endpoints (Rusciano et al., 2017).

The rationale for the use of NTFs as therapeutic agents for glaucoma is their ability to promote RGC survival, regenerate axons, and increase the neuronal function and interconnectivity in such a way that their protection is not limited to preserving the remaining viable RGCs under the glaucomatous condition, but also to foster regeneration of the already lost nerve cells. For example, CNTF delivered by an encapsulated cell technology implant known as the NT-501 device is currently undergoing Phase II clinical trials against the geographic atrophy (age-related macular degeneration) and has been shown to slow down the progression of the vision loss (Zhang et al., 2011). Through this technology, the engineered retinal pigment epithelial cells with encapsulated CNTF were intravitreally implanted into the eyes to give a selective and sustained delivery of CNTF to the RGCs. More trials are conducted using the CNTF implants in POAG patients, although the outcomes are not yet published (Tian et al., 2015). Importantly, unlike CNTF, the human trials failed to show the benefit of the BDNF treatment for repairing the retinal damage. This could be because of the inability of the neurotrophic agents to cross blood ocular barriers. Hence, alternative methods of delivery to bypass the intrinsic biological barriers, should be sought.

It is reasonable to predict that BDNF will require a non-invasive delivery method in humans. Undoubtedly, the unfavourable pharmacokinetic properties (e.g., short half-life and low blood-brain/ocular barrier permeability) are the major hurdles to using BDNF-based therapies in clinical investigations (Houlton et al., 2019). Delivering BDNF to target site remains challenging due to its instability. Poor protein stability is detrimental to the therapeutic efficacy and may elicit potential immunogenic effects associated with the exposure of non-native peptide epitopes, which may act as the adjuvant for BDNF. Ultimately, the biological effects of BDNF will depend on the ability of the drug delivery system to provide a sustained and adequate drug release. With the right approach, it is possible to ameliorate functional axonal regeneration over a short period (Madduri and Gander, 2012). Furthermore, enormous challenges could arise for its human use due to a variety of genetic backgrounds, lifestyles, patterns of physical activity, and age of the patients with variable pathologies and additional medications, which may all affect the overall efficacy. Conclusively, successful NTF delivery requires dosage customization taking into account each of these factors and use of a delivery system optimal for clinical use.

## Adjuvant Therapy to Boost BDNF Signaling

Currently, there is an ongoing effort to achieve the selective activation of BDNF-TrkB *via* the administration of exogenous BDNF or its conjugation with other molecules with a high affinity to TrkB in order to achieve target-specific delivery (Ruiz et al., 2014). One of the approach is the exogenous administration of BDNF with nanoparticles as carriers (Schmidt et al., 2018). In cats with optic nerve damage, combined intravitreal injection of other molecules together with BDNF prolonged the RGC survival (Weber et al., 2010; Weber and Harman, 2013). It is likely that targeting BDNF-TrkB pathways *via* specific upstream or downstream molecules, such as inhibition of the Shp2 phosphatase and GSK-3β activity (Kimura et al., 2016) may prove to be beneficial. To achieve greater therapeutic efficacies, it may also be necessary to combine different compounds that target multiple mechanisms of RGC loss (Konstas et al., 2020). For instance, pharmacological approaches to reduce inflammation and oxidative stress in combination with gene therapy are currently being developed (Katsu-Jiménez et al., 2016; Yin et al., 2020). Although the use of combination therapies has been recommended, there are still no reports on their clinical efficacy.

## Endogenous BDNF Modulation Through Stem Cell Therapy

Stem cell therapy is another approach with the potential to modulate BDNF signaling either by enhancing its production *via* activating multiple neuroprotective pathways or by acting as a nanocarrier. Stem cell-derived RGCs are an ideal treatment option to replace diseased or dead RGCs; however, the complexity of the retinal architecture makes the idea of the cell replacement difficult for functional repair. Alternatively, transplantation of stem cells, such as mesenchymal stem cells (MSCs), also holds a great prospect due to their capacity to secrete exosomes that can serve as extracellular vesicles encapsulating BDNF (Harrell et al., 2019). Interestingly, MSC-derived Exos (MSC-Exos) can survive in the vitreous humor for at least 4 weeks after the intravitreal injection and, because of their nanoscale dimensions, may rapidly reach RGCs to supply them with neurotrophins (Mathew et al., 2019). Accordingly, the intravitreal transplantation of MSCs that were engineered to produce and secrete BDNF at a constant and optimized level were found to preserve the functional and structural integrity of retina in a rat model of chronic ocular hypertension (Harper et al., 2011). Harrell et al. have extensively reviewed the therapeutic potential of the transplantation of MSCs-derived NTFs in glaucoma. The authors suggested that MSCs induced the production of neurotrophins and vasoactive and immunomodulatory factors, which triggered the expansion and regeneration of RGCs in animal models of glaucoma (Harrell et al., 2019). These findings suggest that the emerging role of stem-cell-based therapies as vectors for the delivery of BDNF may be beneficial for the glaucoma treatment. Exciting discoveries are underway by utilizing stem cell therapies, such as the engineered BDNF-producing cells that can be encapsulated (Deng et al., 2016; Pollock et al., 2016) or directly grafted with various moieties (Harper et al., 2011). BDNF gene delivery through recombinant adeno-associated viruses also seems to elicit a sustained increase in the BDNF concentration in the retina and support the survival of RGCs (Osborne et al., 2018). This method seems to hold a great potential for ensuing the BDNF delivery and release to the SC, which the RGCs target. Although viral vector-induced BDNF overexpression in the SC may improve the retrograde transport, it did not improve the BDNF levels in the retina nor did it protect RGCs in glaucomatous animals (Wójcik-Gryciuk et al., 2020).

## BDNF as a Diagnostic Biomarker for Glaucoma

The diagnostic criteria for glaucoma have been extensively debated and specific guidelines are now followed. Currently, diagnosis largely relies on the detection of abnormal changes in the optic disc and the visual field using various tools such as fundoscopy, optical coherence tomography (OCT) and the standard automated perimetry (SAP). However, it is suggested that most of the currently used methods can detect the disease only when 30%–50% of the RGCs have been irreparably lost (Quigley et al., 1992). Nonetheless, early detection of glaucomatous damage is ideal for preventing the progressive loss of RGCs (Aquino and Aquino, 2020). Hence, it is important to look for biomarkers that can predict the onset and/or progression of disease and can be objectively measured and evaluated as an indicator of biological processes in both normal and pathological conditions (Fiedorowicz et al., 2021). For example, an investigation into the relationship between the systemic levels of BDNF and the risk for the development and/or the rate of glaucoma progression may prove beneficial in predicting its possible utility as a biomarker (Oddone et al., 2017). It would also be interesting to explore the relationship of BDNF levels with treatment outcomes and prognosis. This proposition is based on the observations that BDNF levels are considerably lower in the sera, aqueous humors, and lacrimal fluids of patients with early stages of POAG (Shpak et al., 2018). A similar correlation of BDNF levels has been observed in patients with Alzheimer disease (Karege et al., 2005; Beeri and Sonnen, 2016; Eyileten et al., 2021). Since, BDNF is also generated by some non-neural cells, it remains debatable if the source of systemically detected BDNF is in fact the neuronal tissue. Studies have, however, shown that systemic BDNF levels corresponds to BDNF levels in the brain (Mojtabavi et al., 2020). The blood BDNF concentrations across species have been extensively reviewed by Klein et al. (2011). The authors suggest that the blood and plasma BDNF levels very closely reflect BDNF levels in brain tissues. These findings not only give insight into the pathophysiology of the disease but also indicate possible use of systemic BDNF levels as a biomarker for monitoring the onset and progression of neurodegenerative diseases such as glaucoma and Alzheimer disease.

## Challenges and Future Direction

To date, the bonafide intervention for glaucoma, whether it is a pharmaceutical or a surgical procedure, aims to slow down the progression of optic neuropathy and reduce visual field defects by lowering the IOP just enough to maintain good visual functions. Several published clinical trials have explicitly proven that the reduction of IOP could reduce the progression of the visual loss in both early and late stages of the disease. Yet, as it was reported in many cases, patients with excellent IOP readings have had worsening vision despite extensive therapy (Kim and Caprioli, 2018). Even with substantial improvements in therapeutic precision and knowledge on the disease progression, a subset of individuals with glaucoma is prone to aggressive progression, possibly owing to non-IOP-associated factors contributing to the RGC loss (Forchheimer et al., 2011). Besides that, there have been no substantial evidence of non-IOP lowering medications that could alter the glaucoma progression, and none of them could provide neuroprotection to recover the retinal and neural function in clinical trials (Lusthaus and Goldberg, 2019). An analytical review by Storgaard et al. suggests that despite several glaucoma related preclinical and clinical trials in the last 30 years, a successful translations to actual clinical use has not been achieved (Storgaard et al., 2021). The barriers to translate from preclinical into clinical practice may include heterogeneous nature of disease that is difficult to mimic fully in animal models leading to the variability in outcome measures, differences in ocular bioavailability, and the optimal timing of intervention. As opposed to therapeutic intervention after the diagnosis in human, similar interventions in animal studies are employed either before or during induction of disease process. Not to forget, human studies must account for disease variability induced by comorbidities and polypharmacy, which is generally not a component in preclinical studies. Furthermore, development of a formulation to circumvent anatomical and physiological barriers to drug permeation and allow a suitable route of administration, preferably topical, remains challenging.

## Conclusion

RGC degeneration underlies a number of ocular disorders associated with terminal blindness, including glaucoma. Although various pathophysiological mechanisms have been described, deprivation of NTFs is a well-known factor contributing to RGC loss in glaucoma. Among the NTFs, BDNF has widely been investigated for its role in maintaining the integrity of retinal neuronal structure and function. It has a variety of roles extending from embryonic to adult life. The neuroprotective effects of BDNF have been observed by various researchers in preclinical studies; however, it application in clinical setting as monotherapy or adjuvant therapy remains to be explored. This review has highlighted various sources of BDNF, its transport mechanisms within neuronal cells and wide array of its functions. Considering the crucial role of BDNF in physiological functions, manipulations of its cellular pathways to specifically targeting the pathophysiological derangement would be the key to its successful therapeutic application. Additionally, its possible use as a biomarker requires further investigations. Additional medications that can operate concomitantly with the current IOP-lowering medications are desperately needed. However, various forms of glaucoma may require different additional therapies, necessitating an individualized therapeutic approach that considers the patients’ overall health and disease predispositions. Another critical matter is the need to create thoroughly reliable and precise screening procedures, which would enable an early detection of the neuronal injury in glaucoma. The development of clinical tools that are sensitive to retinal structure and changes in function on the scale of months instead of years will profoundly impact clinical trials by shortening their duration and fast-tracking the therapeutic development. This review suggests that BDNF is an exciting target as a biomarker. However, this vast subject still needs further investigation. Therefore, it is important that physicians remain updated on the most recent discoveries, particularly with respect to those highlighting therapeutic benefits of neuroprotective agents.
